# Discrete survival model analysis of a couple’s smoking pattern and outcomes of assisted reproduction

**DOI:** 10.1186/s40738-017-0032-2

**Published:** 2017-02-20

**Authors:** Jose C. Vanegas, Jorge E. Chavarro, Paige L. Williams, Jennifer B. Ford, Thomas L. Toth, Russ Hauser, Audrey J. Gaskins

**Affiliations:** 1 Department of Nutrition, Harvard T.H. Chan School of Public Health, 665 Huntington Ave., 02115 Boston, MA USA; 2 Department of Epidemiology, Harvard T.H. Chan School of Public Health, 665 Huntington Ave., 02115 Boston, MA USA; 3 Channing Division of Network Medicine, Department of Medicine, Brigham and Women’s Hospital and Harvard Medical School, 181 Longwood Ave., 02115 Boston, MA USA; 4 Department of Biostatistics, Harvard School T.H. Chan of Public Health, 665 Huntington Ave., 02115 Boston, MA USA; 5 Vincent Obstetrics and Gynecology, Massachusetts General Hospital and Harvard Medical School, 55 Fruit St, 02114 Boston, MA USA; 6 Department of Environmental Health, Harvard T.H. Chan School of Public Health, 665 Huntington Ave., 02115 Boston, MA USA

**Keywords:** Smoking, Fertility, in vitro fertilization, Assisted reproductive technologies, Reproductive health, Survival analysis

## Abstract

**Background:**

Cigarette smoking has been associated with worse infertility treatment outcomes, yet some studies have found null or inconsistent results.

**Methods:**

We followed 225 couples who underwent 354 fresh non-donor assisted reproductive technology (ART) cycles between 2006 and 2014. Smoking history was self-reported at study entry. We evaluated the associations between smoking patterns and ART success using multivariable discrete time Cox proportional hazards models with six time periods: cycle initiation to egg retrieval, retrieval to fertilization, fertilization to embryo transfer (ET), ET to implantation, implantation to clinical pregnancy, and clinical pregnancy to live birth to estimate hazard ratios (HR) and 95% CIs. Time-dependent interactions between smoking intensity and ART time period were used to identify vulnerable periods.

**Results:**

Overall, 26% of women and 32% of men reported ever smoking. The HR of failing in the ART cycle without attaining live birth for male and female ever smokers was elevated, but non-significant, compared to never smokers regardless of intensity (HR = 1.02 and 1.30, respectively). Female ever smokers were more likely to fail prior to oocyte retrieval (HR: 3.37; 95% CI: 1.00, 12.73). Every one cigarette/day increase in smoking intensity for females was associated with a HR of 1.02 of failing ART (95% CI: 0.97, 1.08), regardless of duration or current smoking status. Women with higher smoking intensities were most likely to fail a cycle prior to oocyte retrieval (HR: 1.07; 95% CI: 1.00, 1.16). Among past smokers, every additional year since a man had quit smoking reduced the risk of failing ART by 4% (HR: 0.96; 95% CI: 0.91, 1.00) particularly between clinical pregnancy and live birth (HR: 0.86; 95% CI: 0.76, 0.96).

**Conclusions:**

Female smoking intensity, regardless of current smoking status, is positively associated with the risk of failing ART cycles between initiation and oocyte retrieval. In men who ever smoked, smoking cessation may reduce the probability of failing ART, particularly between clinical pregnancy and live birth.

**Trial registration:**

NCT00011713. Registered: 27 February 2001.

**Electronic supplementary material:**

The online version of this article (doi:10.1186/s40738-017-0032-2) contains supplementary material, which is available to authorized users.

## Background

Tobacco is among the leading causes of death and disability worldwide [1], with over 6 million people dying of tobacco-related causes per year [2]. It is a common exposure in the US, with smoking reported for approximately 25% of reproductive-aged women, 12% of women during pregnancy [3], and 25% of men aged 25 to 44 years [4]. Cigarette smoke contains about 4000 compounds that have been associated with increased risk of cardiovascular disease, lung disease, cancer [5, 6] and adverse reproductive outcomes [7, 8].

Previous studies have shown that a couple’s likelihood of success when undergoing assisted reproduction varies depending on their smoking history [9]. Female smoking has been associated with poorer ovarian response markers, lower oocyte counts, and lower rates of implantation [10] and live birth [11, 12] following assisted reproductive technology (ART). Male smoking has also been associated with lower live birth rates [12], independent of female smoking. To date, most studies have been limited in their assessment of smoking history, generally comparing “smokers” to “non-smokers” without making clear distinctions among past and never smokers and rarely taking into account smoking intensity, duration or cessation. Furthermore none of the previous studies have utilized methods which allow distinction of smoking effects by stage of the ART cycle, and thus have not always been able to account for early failures, which may have resulted in contradictory findings [13].

Thus it remains unclear whether characteristics of a couple’s smoking history, such as duration and intensity of smoking, the age at smoking initiation, and years since smoking cessation could affect the probability of succeeding at ART. To address this, we evaluated the association of pre-treatment smoking patterns and infertility treatment outcomes in a prospective cohort of couples undergoing ART at the Massachusetts General Hospital (MGH) Fertility Center, using novel statistical methods to allow for a changing effect of smoking on ART failure across time periods within an ART cycle.

## Methods

### Study overview

Participants were couples enrolled in the Environment And Reproductive Health (EARTH) study, an ongoing prospective cohort study started in 2006 aimed at identifying determinants of fertility among couples presenting to the MGH Fertility Center (Boston, MA) [14]. The study was approved by the Institutional Review Boards of MGH and the Harvard T. H. Chan School of Public Health. All participants provided written informed consent.

### Study population

Couples presenting to the MGH Fertility Center were given information about the EARTH Study by the medical team. Study personnel approached couples who met the age requirements (18–46 years for women; 18–55 years for men), and who initially planned to use their own gametes for treatment. Approximately 60% of those contacted participated in the study. Participants were included in the main analysis if they underwent at least one fresh non-donor ART cycle between 2006 and 2014 (*n* = 392). Due to our interest in smoking patterns among couples, we excluded 156 women whose partner had not yet enrolled or elected not to enroll in the EARTH study. Some aspects differed in the excluded couples compared to those included. On average, the female partners from the excluded couples were slightly older (36 vs. 35 years), had higher BMIs (25 vs.24 kg/m^2^), had more pack-years of smoking (1.4 vs. 0.7 pack-years), and were less likely to have completed at least a college education (78% vs. 86%). The cycles from the excluded couples were also more likely to fail prior to embryo transfer (15% vs. 8%). After exclusions, 236 couples who underwent a total of 354 fresh non-donors ART cycles were included in the analysis.

### Smoking assessment

Participants were asked to report whether they had ever smoked cigarettes (defined as smoking at least 1 cigarette/day for a year or at least 20 packs in their lifetime) in a brief staff-administered questionnaire. Individuals reporting a positive smoking history were asked about their age at initiation, current smoking status, smoke inhalation practices, and the brand of cigarette they used (including size, tobacco type, and filter). Couples reporting current use reported what day they had their last cigarette, how many cigarettes they currently smoke per day, average number of cigarettes smoked per day over their years as a current smoker, and whether they had ever quit for 6 months or more and, if yes, how many years they quit. Former users reported how old they were they quit smoking, if they quit in the last year, the date, quantity of cigarettes smoked per day during the period they smoked, and if before they quit there were periods (≥6 months) when they didn’t smoke. The total smoking duration was calculated in current users by subtracting the age of smoking initiation from their current age. In former smokers we subtracted the age of smoking initiation from their age on cessation. For both we subtracted their interim cessation periods from their total smoking duration. Pack-years of smoking was calculated as: $\frac{Average\kern0.24em  number\; of\; cigarettes\; smoked\; a\; day}{20\; cigarettes\; per\; pack}\times Total\; smoking\; duration(years)$ [15]. Questions on the use of other tobacco products (i.e. cigars, pipes, chewing tobacco and snuff or dip tobacco) were included on the questionnaire, but due to their infrequent use in our sample (<2% for cigars and <1% for the rest), only cigarettes smoking was considered as an exposure in our analyses.

### Covariates

At enrollment, a brief nurse-administered questionnaire was used to collect data on demographic characteristics, medical history, and lifestyle factors. A trained research study staff member measured each participant’s height and weight.

### ART Outcomes

Women underwent a pretreatment cycle of oral contraceptives for 2–5 weeks to suppress ovulation before their ART cycles, unless contraindicated. On day 3 of induced menses, patients began controlled ovarian stimulation. Women underwent one of three stimulation protocols as clinically indicated: 1) luteal-phase GnRH agonist protocol; 2) follicular-phase GnRH-agonist/flare protocol; or 3) GnRH-antagonist protocol. Patients were monitored during gonadotropin stimulation for serum estradiol and endometrial thickness through 2 days before egg retrieval. Human chorionic gonadotrophin was administered 36 h before the scheduled egg retrieval procedure to induce ovulation. Details of oocyte retrieval have been previously described [16]. Embryologists classified oocytes as germinal vesicle, metaphase I, metaphase II (MII), or degenerated. Following retrieval, couples underwent conventional insemination or intracytoplasmic sperm injection (ICSI) for fertilization. We defined successful implantation as a serum β-hCG level >6 mIU/mL 15–20 days after egg retrieval, clinical pregnancy as the presence of an intrauterine pregnancy in ultrasound, and live birth as the birth of a neonate at or after 24 weeks of gestation. All clinical information was abstracted from electronic medical records.

### Statistical analysis

Both male and female participants were classified into three categories: never, former, and current smokers according to self-reported smoking status. We then cross-classified couples into four categories according to their ever smoking status. We evaluated the effect of smoking intensity using the variables average cigarettes/day and total years of smoking as continuous exposures. Descriptive statistics were calculated for demographic and reproductive characteristics according to current smoking status prior to ART treatment. A Chi-square test or Fisher’s exact test was used to test for differences across categories for discrete variables and the Kruskal-Wallis test for differences across categories for continuous variables.

Multivariable Cox proportional hazards models for discrete survival time were used to estimate the hazard ratios (HRs) and 95% confidence intervals (CIs) for the associations between smoking patterns and risk of failing ART prior to live birth, as proposed by Maity [17] and Missmer [18]. In all of these models, a robust sandwich covariance estimate was used to account for the multiple cycles per woman. HRs estimate the odds of failing an ART cycle at any point, conditional on not failing at an earlier moment during the same cycle. Women were considered at risk of failing ART for the duration of their initiated cycle until their specific point of failure. There were six periods during which women could fail a cycle: between (1) cycle initiation and oocyte retrieval, (2) retrieval and fertilization, (3) fertilization and embryo transfer (ET), (4) ET and implantation, (5) implantation and clinical pregnancy, and (6) between clinical pregnancy and live birth. Estimated probabilities of survival for the average women in our cohort (according to mean or reference levels for covariates other than smoking) were used to construct survival curves across these time points.

Confounding was evaluated through directed acyclic graphs based on prior knowledge and descriptive statistics in the study population. The multivariable models were adjusted for age, BMI, protocol, educational level and partner’s smoking history (never or ever smoker). We centered smoking intensity, years of smoking duration and pack-years by the whole sample’s mean for continuous analyses. For analysis of smoking history (ever vs. never smoker), smoking status (never, former and current smoker) and couple’s cross-classifications, we further adjusted by centered pack-years. We analyzed as continuous variables smoking intensity and duration in the entire population years since smoking cessation and age at smoking initiation among ever smokers (58 women and 71 men). Never smokers were assigned a value of 0 average cigarettes/day and 0 years of total smoking for the continuous analyses. Since in Massachusetts insurance companies require current smokers to have cotinine levels below the non-smoker reference value for 2 months to provide authorization for IVF coverage, we assigned a cessation time of 2 months for current smokers. All results are presented as HRs for a 1 unit increase in the continuous exposure. We further adjusted the models for smoking intensity (cigarettes/day) and smoking duration (years) using a binary variable for self-reported current smoking status. Models for smoking cessation and age at initiation were further adjusted for pack-years. The model of age at smoking initiation was also further adjusted for a binary variable for self-reported current smoking-status. To evaluate whether there were specific stages of the cycle which were more vulnerable to the effects of smoking, we fit multivariate models with time-dependent interaction between the discrete time periods and smoking exposures.

We also ran a variety of sensitivity analysis to address potential issues related to misclassification of the exposure, selection bias into the study, and model fitting. In accordance with a prior methodological paper on smoking and lung cancer [19], we conducted a sensitivity analysis re-classifying the participant’s self-reported smoking status according to their time of cessation in three discrete categories: never smokers (self-reported never smokers), former smokers (self-reported former smokers with > 2 years of smoking cessation) and “*clinically active smokers*” (self-reported current smokers and self-reported former smokers with ≤ 2 years of smoking cessation). Also, in order to assess the possibility of selection bias in our sample (due to the exclusion of women who did not have a partner enrolled), we conducted a sensitivity analysis including all woman regardless of whether their partner was enrolled and analyzed the association between smoking history and probability of failing ART without adjusting for the male partner’s smoking history. Finally, we performed an analysis limited to the couple’s first cycle to address the potential limitation of this model to account for the imbalanced number of cycles per couple.

To illustrate the possible effect of survival bias, we used generalized linear mixed models with random intercepts to analyze the associations between smoking patterns and oocyte yield (Poisson distribution and log link); endometrial wall thickness and peak serum estradiol at trigger day (binomial distribution and log link) among women who underwent oocyte retrieval, and probability of live birth per initiated cycle and per embryo transfer (binary distribution and logit link). These results are presented as population marginal means adjusted for covariates. All analyses were conducted using the Statistical Analysis System Software package SAS 9.4 (SAS Institute Inc., Cary, NC) and considered two-sided significance levels < 0.05 as statistically significant.

## Results

Our sample consisted mostly of non-Hispanic Caucasians (86% of women; 88% of men) with a mean age of 34.8 and 36.6 years for female and male participants, respectively. About 26% of women were self-reported ever smokers (23% former and 3% current smokers); 32% of men were ever smokers (24% former and 7% current smokers). On average, women who ever smoked started smoking when they were 17 years, smoked 6 cigarettes/day for 9 years, and if they had quit, they did so 7.5 years before cycle initiation. Similarly, men who ever smoked started smoking when they were an average of 18 years old, and on average smoked 9 cigarettes per day for 12 years, and if they had quit, they did so 6 years before cycle initiation. Men and women who were current smokers were more likely to have a partner who currently smoked and were less likely to have a college education compared to never smokers (Tables 1 and 2). Current smokers also reported smoking for longer durations compared to former smokers. Current male smoker were more likely to have undergone reproductive tract surgery prior cycle initiation. Current female smokers were more likely to have their first cycle cancelled prior to embryo transfer and had higher baseline FSH levels.

**Table 1:** Baseline characteristics of 225 couples from the EARTH study in relation to female smoking status

	Female Smoking Status			*P-value* ^*a*^
	Never smoker	Former smoker	Current smoker	
Number of couples, n (%)	167 (74.2)	51 (22.7)	7 (3.1)	<0.001
Total ART cycles, n (%)	249 (70.3)	93 (26.3)	12 (3.4)	<0.001
*Female Characteristics* ^*b*^				
Age, years	35 (32, 38)	34 (33, 37)	34 (29, 37)	0.89
Body Mass Index, kg/m^2^	22.6 (20.4, 26.3)	22.7 (21.5, 24.7)	25.7 (23.8, 28.2)	0.11
At least college education, n (%)	148 (88.6)	39 (76.5)	5 (71.4)	0.04
Non-Hispanic Caucasian, n (%)	143 (85.6)	43 (84.3)	7 (100)	0.74
Pack-years	NA	1.6 (0.5, 4.6)	2.4 (1.0, 4.2)	0.65
Total smoking duration, years	NA	9 (5, 13)	14 (8, 16)	0.08
Average cigarettes smoked	NA	4.0 (1.5, 10.0)	5.0 (1.5, 7.0)	0.89
Age of smoking initiation, years	NA	17 (15, 18)	18 (18, 20)	0.04
Total Smoking cessation, years	NA	8.0 (5.0, 12.0)	0.2 (0.2, 0.2)	<0.001
Exposed to passive smoking, n (%)	30 (18.0)	14 (27.5)	2 (28.6)	0.23
*Male Partner Characteristics*				
Partner age, years	36 (33, 40)	35 (33, 40)	39 (35, 41)	0.40
Partner body mass index, kg/m^2^	26.9 (24.2, 29.3)	26.9 (24.5, 29.3)	27.9 (25.1, 28.3)	0.97
Partner former smoker, n (%)	38 (22.8)	16 (31.4)	1 (14.3)	0.44
Partner current smoker, n (%)	8 (4.8)	4 (7.8)	4 (57.1)	<0.001
*Baseline Reproductive Characteristics*				
Previous IUI, n (%)	71 (42.5)	19 (37.3)	2 (28.6)	0.70
Previous IVF, n (%)	40 (24.0)	8 (15.7)	1 (14.3)	0.51
ICSI, n (%)	83 (49.7)	31 (60.8)	4 (57.1)	0.37
Infertility diagnosis, n (%)				0.47
Male factor	55 (32.9)	20 (39.2)	4 (57.1)	
Diminished ovarian reserve	9 (5.9)	3 (5.9)	1 (14.3)	
Endometriosis	10 (6.0)	3 (5.9)	0 (0.0)	
Ovulatory dysfunction	15 (9.0)	5 (9.8)	0 (0.0)	
Tubal causes	11 (6.6)	8 (15.7)	1 (14.3)	
Uterine causes	3 (1.8)	0 (0.0)	0 (0.0)	
Unexplained	64 (38.3)	12 (23.5)	1 (14.3)	
Initial treatment protocol, n (%)				0.69
Antagonist	21 (12.6)	5 (9.8)	1 (14.3)	
Flare	17 (10.2)	8 (15.7)	0 (0.0)	
Luteal phase agonist	129 (77.3)	38 (75.5)	6 (85.7)	
Day 3 FSH levels, IU/L	6.6 (5.8, 8.0)	7.3 (6.0, 8.8)	8.1 (7.0, 8.3)	0.16
E2 Trigger levels, pmol/L	2126 (1632, 2639)	1828 (1459, 2532)	1798 (1540, 1953)	0.17
Day of embryo transfer, n (%)				0.01
No transfer	11 (6.6)	4 (7.8)	2 (28.6)	
Day 2	4 (2.4)	5 (2.2)	0 (0.0)	
Day 3	79 (47.6)	29 (56.9)	1 (14.3)	
Day 5	72 (43.4)	13 (25.5)	4 (57.1)	
Number of embryos transferred, n (%)				<0.001
No transfers	11 (6.6)	4 (7.8)	2 (28.6)	
1 embryo	42 (25.3)	2 (3.9)	2 (28.6)	
2 embryos	81 (48.8)	41 (80.4)	3 (42.7)	
3 or more embryos	32 (19.3)	4 (7.8)	0 (0.0)	

^a^From Chi-square or Fisher test for discrete variables and Kruskal-Wallis for continuous variables

^b^Continuous variables are presented as median (25^th^ percentile, 75^th^ percentile) while categorical variables are presented as number of women (percent)

*Abbreviations*: *ART* Assisted reproductive technology, *BMI* Body mass index, *E2* estradiol, *FSH* follicle stimulating hormone, *IVF* In-vitro fertilization, *ICSI* Intracytoplasmic sperm injection, *IUI* intrauterine sperm insemination

**Table 2:** Baseline characteristics of 225 couples from the EARTH study in relation to male smoking status

	Male Smoking Status			*P* –value
	Never smoker	Former smoker	Current smoker	
Number of couples, n (%)	154 (68.4)	55 (24.4)	16 (7.1)	<0.001
Total ART cycles, n (%)	235 (66.4)	89 (25.1)	30 (8.5)	<0.001
*Male Characteristics* ^b^				
Age, years	35 (32, 40)	37 (34, 40)	37 (33, 39)	0.20
BMI, kg/m^2^	26.8 (24.3, 29.1)	27.4 (24.3, 30.0)	25.6 (23.4, 28.3)	0.33
At least college education, n (%)	111 (72.1)	33 (60.0)	7 (56.3)	0.15
Non-Hispanic Caucasian, n (%)	137 (89.0)	48 (87.3)	13 (81.3)	0.58
Pack-years	NA	3.3 (1.1, 7.3)	7.0 (3.3, 8.7)	0.07
Total smoking duration, years	NA	10 (5, 14)	17 (14, 19)	0.001
Average cigarettes smoked	NA	6.0 (3.0, 12.0)	9.3 (4.8, 11.3)	0.68
Age of smoking initiation, years	NA	18 (16, 20)	16 (15, 20)	0.22
Total Smoking cessation, years	NA	4.0 (1.5, 7.8)	0.2 (0.2, 0.2)	<0.001
Exposed to passive smoking, n (%)	24 (15.6)	14 (25.5)	7 (43.8)	0.01
*Male Reproductive History*				
History of cryptorchidism, n (%)	10 (6.5)	2 (3.7)	2 (12.5)	0.42
History of varicocele, n (%)	16 (10.4)	4 (7.3)	2 (12.5)	0.90
History of reproductive surgery, n (%)	31 (20.1)	8 (14.6)	7 (43.8)	0.04
*Female Partner Characteristics*				
Partner age, years	34 (32, 38)	35 (33, 38)	36 (33, 39)	0.47
Partner BMI, kg/m^2^	22.2 (20.7, 25.4)	23.8 (21.3, 27.7)	24.8 (20.5, 29.2)	0.11
Partner former smoker, n (%)	31 (20.1)	16 (29.1)	4 (25.0)	0.38
Partner current smoker, n (%)	2 (1.3)	1 (1.8)	4 (25.0)	<0.001
*Baseline Reproductive Characteristics*				
Previous IUI, n (%)	67 (43.5)	18 (32.7)	7 (43.8)	0.37
Previous IVF, n (%)	34 (77.9)	11 (20.0)	4 (25.0)	0.90
ICSI, n (%)	82 (53.3)	27 (49.1)	9 (56.3)	0.83
Infertility diagnosis, n (%)				0.53
Male factor	55 (35.7)	16 (29.1)	8 (50.0)	
Diminished ovarian reserve	8 (5.2)	3 (5.5)	2 (12.5)	
Endometriosis	6 (3.9)	6 (10.9)	1 (6.3)	
Ovulatory dysfunction	13 (8.4)	7 (12.7)	0 (0.0)	
Tubal Causes	13 (8.4)	5 (9.1)	2 (12.5)	
Uterine Causes	2 (1.3)	1 (1.8)	0 (0.0)	
Unexplained	57 (37.0)	17 (30.9)	3 (18.8)	
Initial treatment protocol, n (%)				0.05
Antagonist	16 (10.4)	5 (9.1)	6 (37.5)	
Flare	18 (11.7)	5 (9.1)	2 (12.5)	
Luteal phase agonist	120 (77.9)	45 (82.8)	8 (50.0)	
Day 3 FSH levels, IU/L	6.7 (5.8, 8.0)	6.6 (5.8, 8.2)	8.2 (7.3, 9.5)	0.004
E2 Trigger levels, pmol/L	2076 (1560, 2678)	2145 (1632, 2570)	1761 (1225, 2311)	0.27
Day of embryo transfer, n (%)				0.90
No transfer	12 (7.8)	2 (7.3)	1 (6.3)	
Day 2	7 (4.6)	1 (1.8)	1 (6.3)	
Day 3	71 (46.4)	29 (52.7)	9 (56.3)	
Day 5	63 (41.2)	21 (38.2)	5 (31.3)	
Number of embryos transferred, n (%)				0.90
No transfer	12 (7.8)	2 (7.3)	1 (6.3)	
1 embryo	34 (22.2)	8 (14.6)	4 (25.0)	
2 embryos	83 (54.3)	34 (61.8)	8 (50.0)	
3 or more embryos	24 (15.7)	9 (16.4)	3 (18.8)	

^a^From Chi square or Fisher test for discrete variables and Kruskal-Wallis for continuous variables

^b^Continuous variables are presented as median (25^th^ percentile, 75^th^ percentile) while categorical variables are presented as number of women (percent)

*Abbreviations*: *ART* Assisted reproductive technology, *BMI* Body mass index, *E2* estradiol, *FSH* Follicle stimulating hormone, *IVF* In-vitro fertilization, *ICSI* Intracytoplasmic sperm injection, *IUI* Intrauterine sperm insemination

Smoking status of the female partner, the male partner, and the cross-classified couple smoking status were unrelated to the risk of cycle failure (Table 3). The HR of failing ART for female ever smokers compared to never smokers was 1.30 (95% CI: 0.84, 2.02; *P-Value* =0.24) and estimated HRs remained similar even after accounting for smoking intensity and duration. When evaluating the effects of smoking during specific periods of potential ART failure, ever female smokers were marginally more likely to fail prior to oocyte retrieval (HR: 3.57; 95% CI: 1.00, 12.73; *P-Value* = 0.05) but not during other periods of the ART cycle. In the self-reported current smoking status analysis, the HR of failing ART for former and current female smokers was 1.23 (95% CI: 0.79, 1.91; *P-Value* = 0.35) and 2.41 (95% CI: 0.62, 9.39; *P-Value* = 0.20) when compared to never smokers. Similarly, current smokers (HR: 6.0; 95% CI: 0.96, 37.07; *P-Value* = 0.05) and former smokers (HR: 2.05; 95% CI: 0.50, 8.34; *P-Value* = 0.32) were more likely to fail prior to oocyte retrieval compared to never smokers but this association was not significant for the other ART time points and only borderline significant for current smokers. Women whose male partners were former and current smokers had no increased risk of failing ART when compared to never smokers. In addition, couple classifications in which either or both of the partners ever smoked, as compared to couples in which neither partner ever smoked, did not have an increase in risk of cycle failure.

**Table 3:** Estimated cumulative probabilities of live birth and corresponding hazard ratios of failing at ART by discrete smoking patterns (EARTH study, *N* = 225 couples, 354 ART cycles)

	Number of Couples/Cycles	Estimated cumulative probability of live birth, per 100 cycles^a^	Adjusted Hazard Ratio (95% CI)^b^	*P*-Value^b^
*Female Smoking History*				0.24
Never Smoker	167/249	54.2 (45.8, 64.0)	1.00 (REF)	
Ever Smoker	58/105	45.0 (32.8, 61.8)	1.30 (0.84, 2.02)	
*Female Smoking Status*				0.35
Never Smoker	167/249	54.1 (45.7, 63.9)	1.00 (REF)	
Former Smoker	51/93	46.9 (34.5, 63.9)	1.23 (0.79, 1.91)	
Current Smoker	7/12	22.7 (5.6, 92.0)	2.43 (0.64, 9.39)	
*Male Smoking History*				0.93
Never Smoker	154/235	54.0 (46.6, 68.4)	1.00 (REF)	
Ever Smoker	71/119	53.2 (43.1, 65.7)	1.02 (0.64, 1.63)	
*Male Smoking Status*				0.75
Never Smoker	154/235	54.3 (45.7, 64.6)	1.00 (REF)	
Former Smoker	55/89	56.4 (44.6, 71.5)	0.94 (0.55, 1.61)	
Current Smoker	16/30	46.7 (30.6, 72.7)	1.25 (0.69, 2.25)	
*Couples Ever Smoking Status*				0.59
Female and Male Never Smokers	121/172	54.7 (45.7, 65.4)	1.00 (REF)	
Female Never Smoker, Male Ever Smoker	46/77	50.7 (38.4, 67.0)	1.12 (0.69, 1.84)	
Female Ever Smoker, Male Never Smoker	33/63	42.2 (28.7, 62.0)	1.43 (0.85, 2.41)	
Female and Male Ever Smokers	25/42	47.5 (31.8, 70.8)	1.24 (0.62, 2.46)	

All estimates were calculated from a discrete time Cox proportional Hazard models adjusting for female age (centered continuous) and BMI (continuous); induction protocol (categorical); female educational status (categorical); centered pack-years history (centered continuous) and partner’s smoking status (categorical)

^a^Estimates of cumulative probability of not failing ART cycle are given for the average couple in our cohort (e.g. female age (34.8 years) and BMI (23.8 kg/m2); induction protocol (luteal agonist); female educational status (more than college); centered pack-years history (0.93 packs-years for women; 2.47 packs-years for men) and partner’s smoking status (never smoker)

^b^ *P*-Value is testing for any difference across categories of exposure

For the intensity and duration analyses (Table 4), a 1 cigarette/day increase in female smoking intensity was associated with a 7% increased risk of failing at ART (HR = 1.07, 95% CI: 0.97, 1.08; *P-Value* = 0.45) after further adjustment for smoking duration and current smoking status; while a 1 year increase in smoking duration yielded a HR of 1.01 (95% CI: 0.97, 1.05 *P-Value* = 0.61) after further adjustment for smoking intensity and current smoking status. Greater intensity of female smoking was associated with a borderline higher risk of failing a cycle prior to oocyte retrieval (HR: 1.07; 95% CI: 1.00, 1.16; *P-Value* = 0.07); In contrast smoking duration was not significantly related to the probability of failing ART during any period of the ART cycle. Neither time of cessation nor age of smoking initiation was significantly associated with the risk of failing ART (Table 4). We found a borderline significant protective effect of smoking cessation in ever-smoker males; for every additional year since a male smoker quit, the risk of failing during an ART cycle decreased by 4% (HR: 0.96; 95% CI: 0.91, 1.00; *P-Value* <0.05) regardless of smoking duration and intensity. This association was particularly pronounced for the time period between clinical pregnancy and live birth (HR: 0.86; 95% CI: 0.76, 0.96; *P-Value* = 0.01).

**Table 4:** Hazard ratios for failing ART by continuous smoking patterns (EARTH study, *N* = 225 couples, 354 ART cycles)

Smoking Pattern	Adjusted Hazard Ratio (95% CI)	*P*-Value
*Female Smoking Intensity and Duration* ^*a*^		
Per 1 additional cigarette per day	1.02 (0.97, 1.08)	0.45
Per 1 additional year of smoking	1.01 (0.97, 1.05)	0.68
*Female Smoking Cessation* ^*b*^		
Per 1 additional year of smoking cessation	1.01 (0.95, 1.08)	0.69
*Female Age of Smoking Initiation* ^*c*^		
Per 1 additional year later smoking initiation	0.90 (0.79, 1.02)	0.11
*Male Smoking Intensity and Duration* ^*a*^		
Per 1 additional cigarette per day	0.99 (0.97, 1.01)	0.36
Per 1 additional year of smoking	1.01 (0.99, 1.04)	0.25
*Male Smoking Cessation* ^*b*^		
Per 1 additional year of smoking cessation	0.96 (0.91, 1.00)	0.05
*Male Age of Smoking Initiation* ^*c*^		
Per 1 additional year later smoking initiation	1.04 (0.91, 1.19)	0.58

^a^Included all couples and cycles (*N* = 354 cycles). Data is presented as the cumulative hazard ratio for failing ART at any point per 1 unit increase of smoking intensity (continuous, centered average cigarettes/day) and total years of smoking (continuous, centered years). The model was adjusted for female age and BMI, protocol, educational status, current smoking status and partner’s smoking status

^b^Limited to ever smokers only (*N* = 105 cycles for women; *N* = 119 cycles for men). Data is presented as the cumulative hazard ratio for failing ART at any point per 1 unit increase of smoking cessation (centered, continuous years). The model was adjusted for female age and BMI, protocol, educational status, pack-years history and partner’s smoking status

^c^Limited to ever smokers only (*N* = 105 cycles for women; *N* = 119 cycles for men). Data is presented as the cumulative hazard ratio for failing ART at any point per 1 unit increase of smoking age at initiation (centered, continuous years). The model was adjusted for female age and BMI, protocol, educational status, pack-years history, current smoking status and partner’s smoking status

Reclassification of smoking status by taking into account time since smoking cessation did not significantly alter our results. For instance, compared to never smokers, former smokers had a 23% increase in risk (HR = 1.23,95% CI: 0.78, 1.95; *P-Value* = 0.37) while “clinically active” smokers had a HR of 1.62 (95% CI: 0.70, 3.78; *P-Value* = 0.37) of failing at ART; similar to the main analysis, the increased risk of failing at ART was predominantly prior to oocyte retrieval (HR: 2.47; 95% CI: 1.27, 4.78; *P-Value* = 0.01) for “clinically active” smokers. The sensitivity analysis including the women without a male partner enrolled in the study provided consistent results to those seem in the main sample. Specifically, female ever smokers had a borderline significant HR of 1.38 (95% CI: 0.99, 1.92; *P-Value* = 0.06) compared to never smokers; also, this increased risk was significantly higher for failing prior to oocyte retrieval (HR: 2.45; 95% CI: 1.12, 5.36; *P-Value* = 0.02) and it was also significant between embryo transfer and implantation (HR: 1.63; 95% CI: 1.03, 2.58; *P-Value* = 0.04). An increase of 1 cigarette/day produced an overall HR of 1.01 (95% CI: 0.97, 1.06; *P-Value* = 0.65) for failing at ART and the HR was not significantly different according to the particular moment of the cycle. Finally, sensitivity analyses limited to the couple’s first cycle produced results that were consistent with the main analysis, although with loss of significance given the reduction in power: female ever smokers had an HR of 1.34 (95% CI: 0.76, 2.34; *P-Value* = 0.31) of failing ART compared to never smokers and this increased risk was more pronounced prior to oocyte retrieval (HR: 3.00; 95% CI: 0.56, 16.08; *P-Value* = 0.20) although these increases were not statistically significant.

Given the observed relation between female smoking and early ART failures, we analyzed the associations between smoking patterns and oocytes counts among women with successful egg retrieval using conventional methods to illustrate the possible effect of survival bias. In these models, self-reported current smokers appeared to have a higher number of total and MII phased oocytes retrieved than former and never smokers even after multivariable adjustment, although these differences did not attain statistical significance (Additional file 1: Table S1); however, 16.7% of cycles from self-reported current smokers could not be included in this analysis because they failed prior to retrieval compared to only 2.0% of cycles from never smokers and 6.5% from former smokers (Additional file 1: Table S1). We did not observe associations with endometrial thickness or peak estradiol levels. When we analyzed the associations between smoking patterns and live birth following ART among the cycles of women undergoing an embryo transfer and all initiated cycles, the predicted probability of live birth was the lowest for current smokers in both cases. However, the differences by smoking status for women undergoing an embryo transfer were attenuated compared to those observed taking into consideration all initiated cycles (Additional file 2: Table S2). This was to be expected as 25% of cycles from self-reported current smokers failed prior to ET compared to only 6.8% of cycles from never smokers.

## Discussion

In a prospective cohort of couples undergoing infertility treatment with ART, we found that the female smoking intensity was related to a higher risk of cycle failure prior to oocyte retrieval, even after accounting for duration of smoking and current smoking status. Our results suggest that the effects of smoking on ovarian response to controlled ovarian stimulation may persist after smoking cessation. We also found a borderline protective effect for male smoking cessation and cycle failure between clinical pregnancy and live birth in former smokers. Our conventional analysis highlighted the possibility that studies ignoring failures prior to oocyte retrieval for intermediate outcomes and failures prior to embryo transfer for clinical outcomes may be susceptible to survival bias, possibly explaining some inconsistencies in the literature of the effects of smoking on human fertility, such as a higher number of oocytes or no differences in clinical outcomes comparing current to never smokers [13].

Despite the low prevalence of current smoking in our cohort, our results do suggest that female smoking is detrimental to overall ART success. These results are in accordance with previous cohort studies and meta-analyses which reported lower probabilities of implantation, clinical pregnancy and live birth following ART in smokers compared to never smokers [9, 12, 20, 21]. Similar to our findings, many of these previous studies also found that female smokers were more likely to fail before an embryo transfer [12, 21, 22]. The higher odds of failing between cycle initiation and oocyte retrieval could imply that cigarette smoke disrupts the response to controlled ovarian hyper stimulation protocols or disrupts the ovary itself. This mechanism could be related to findings in animal models, in which cigarette smoke has been found to alter the serum levels of sex hormones and gametogenesis [23, 24]. Although this mechanism accounts only for contemporaneous exposures there seems to be a relation between lower fecundity and cumulative smoking exposure in epidemiological studies [25]. Other epidemiological studies have shown that current smokers were more likely to have worse ovarian stimulation outcomes compared to never smokers [26, 27]. Our findings also suggest an increased risk of failure at implantation that may have been underpowered in our study, as further evidenced by the results of the second sensitivity analysis which included a larger sample of women.

Not all epidemiological studies have found negative associations between smoking and ART success [13, 28]. Although differences in study design and power may have influenced these results, these studies also tended to analyze clinical outcomes only among cycles reaching the point of embryo transfer. Due to the effects of smoking on early ART outcomes (e.g. response to controlled ovarian stimulation), restricting analyses to only cycles with embryo transfer creates a selection bias in which only the women who survived until then are included, biasing the observed burden of smoking downwards [29] even despite the well-recognized associations between smoking and miscarriage, stillbirth and neonatal death [30]. When we analyzed the association between smoking patterns and live birth per embryo transfer (as opposed to per initiated cycle; Additional file 2: Table S2), we illustrated how this issue may be at play. We also found paradoxical results when we evaluated the association between smoking patterns and oocyte counts among women with successful egg retrieval, as current smokers appeared to yield as many oocytes compared to former and never smokers. However, this association appeared to be largely driven by the 12% of cycles from smokers that failed prior to egg retrieval which was more than double the percentage of failures from never (2.0%) and former smokers (6.3%). Future observational studies should use the appropriate statistics to account for these effects, particularly when exposures may exert effects early in gestation.

Other than a possible beneficial effect of smoking cessation among male ever-smokers, we did not observe any significant associations when studying male smoking patterns and ART outcomes. Nevertheless, male smoking has been associated with lower semen quality parameters [31] and live birth rates [22] in other epidemiological studies. While our analyses were restricted by power, the fact that former male smokers seemed to more quickly benefit from smoking cessation could be explained by the biology of sperm division and differentiation. The mechanism for the observed reduction in cycle failure between clinical pregnancy and live birth due to paternal smoking cessation could be due to lower maternal exposure to second hand smoking and/or a direct paternal exposure [32], although the exact mechanisms remain obscure.

Our study has certain limitations. Due to our design, it may not be possible to extrapolate our results to the general population. However, our cohort of men and women are similar to the population of infertility patients nationwide [33] suggesting that our results might be representative of other couples undergoing ART. While we lacked specific information on the women who declined to participate in the EARTH study, we had a similar smoking prevalence as other ART cohorts in the US [21]. Furthermore, in a previous study on semen quality from MGH, we found that men who refused to participate were comparable to the men who did participate in terms of baseline characteristics [34]. This suggests that the likelihood of selection bias in our study (e.g. smoking men and women being less likely to participate) is low. The women excluded had different baseline characteristics than the included sample; however, our results were similar after performing a sensitivity analysis among all EARTH women and, in fact, some associations gained significance with this increase in power. Another generalizability concern is that in Massachusetts, insurance companies will generally not allow current smokers to have authorization for IVF coverage until cotinine levels are below the non-smoker reference value for 2 months. Therefore self-reported current smokers might not have been smoking for the past two months, which might have attenuated the magnitude of associations towards the null. To address this we assumed a conservative cessation time of at least 2 months for current smokers when modeling time of smoking cessation a continuous exposure. Smoking history was assessed through self-report in our population as opposed to more objective markers like cotinine. While it is possible there was some misclassification of current smokers due to its subjectivity, a person’s self-report of smoking is considered reliable [35]. To minimize the possible effect of passive smoking, we adjusted for the partner’s smoking history. Although our survival models can account for multiple cycles per women, they are unable to account for the imbalance in number of cycles per couple (e.g. women with more cycles are more likely to fail) [17], yet even when we restricted the analysis to just the first cycle per couple the results were similar. Residual and unmeasured confounding is always possible as this was an observational cohort study; however, we attempted to minimize this by adjusting for number of demographic and lifestyle factors and focusing on a couple analyses. Our main limitation was the small numbers of former and current smokers, reducing our power to detect associations. Also, the subjects who did smoke reported being mostly “light” or “moderate” smokers in clinical terms. Our study strengths include our prospective design, our detailed assessment of smoking history, and our ability to account for many confounders including the partner’s smoking.

## Conclusions

Female smoking intensity was positively associated with the risk of failing in ART cycles between cycle initiation and oocyte retrieval. Among former male smokers, every additional year of smoking cessation was associated with a slightly decreased risk of failing at ART between clinical pregnancy and live birth. Future studies should further investigate the long-term effects of smoking intensity on reproductive potential and take care to use the appropriate analytical approaches to account for failures at early stages of reproduction to reduce the likelihood of survivor bias.

## Additional files

Additional file 1: Table S1. Adjusted mean oocyte yield by smoking patterns among couples with successful egg retrieval. (EARTH Study, *N* = 225 couples, 354 ART cycles). (DOCX 17 kb)

Additional file 2: Table S2. Adjusted estimated probability of cycles ending in live birth by smoking patterns (EARTH Study, *N* = 225 couples, 354 ART cycles). (DOCX 17 kb)

## Abbreviations

95% CI: 95% confidence interval
ART: Assisted reproductive technology
BMI: Body mass index
EARTH: Environment And Reproductive Health study
ET: Embryo transfer
HR: Hazard ratio
ICSI: Intracytoplasmic sperm injection
IVF: In-vitro fertilization
MGH: Massachusetts General Hospital
MII: Metaphase II oocytes
