# Supplementary material for: Discrete survival model analysis of a couple’s smoking pattern and outcomes of assisted reproduction
Source: Fertil Res Pract. 2017 Feb 20;3:5. doi: 10.1186/s40738-017-0032-2 (PMC5416813; doi:10.1186/s40738-017-0032-2)
Supplement: Supplementary file 1 — Adjusted mean oocyte yield by smoking patterns among couples with successful egg retrieval. (EARTH Study, N = 225 couples, 354 ART cycles). (DOCX 17 kb) [file 40738_2017_32_MOESM1_ESM.docx]

**Supplemental Table 1.** Adjusted mean oocyte yield by smoking patterns among couples with successful egg retrieval. (EARTH Study, N=225 couples, 354 ART cycles).

|  | **Women/Cycles** | **% of cycles failing prior to oocyte retrieval** | **Peak serum Estradiol level at trigger, pmol/L ^a^** | **Endometrial wall thickness, mm ^a^** | **Total oocyte yield, n ^b^** | **MII oocytes Yield, n ^b^** |
| --- | --- | --- | --- | --- | --- | --- |
|  |  |  | **Adjusted mean (95% CI)** | | | |
| *Female Smoking History* |  |  |  |  |  |  |
| Never Smoker | 167/249 | 2.0% | 2191 (2033, 2349) | 10.2 (9.8, 10.7) | 12.0 (11.1, 13.1) | 10.2 (9.3, 11.1) |
| Ever Smoker | 58/105 | 7.6% | 1974 (1717, 2230) | 10.2 (9.5, 10.9) | 11.6 (10.1, 13.3) | 9.9 (8.5, 11.4) |
| *Female Smoking History* |  |  |  |  |  |  |
| Never Smoker | 167/249 | 2.0% | 2193 (2035, 2352) | 10.2 (9.8, 10.7) | 12.1 (11.1, 13.2) | 10.3 (9.4, 11.2) |
| Former Smoker | 51/93 | 6.5% | 1962 (1700, 2223) | 10.2 (9.5, 10.9) | 11.3 (9.8, 13.0) | 9.6 (8.3, 11.1) |
| Current Smoker | 7/12 | 16.7% | 2109 (1473, 2746) | 10.0 (8.3, 11.8) | 15.0 (10.7, 21.0) | 13.4 (9.5, 18.9) |
| *Couples Ever Smoking Status* |  |  |  |  |  |  |
| Female and Male Never Smokers | 121/172 | 2.3% | 2187 (2014, 2360) | 10.2 (9.7, 10.7) | 11.6 (10.6, 12.7) | 9.9 (9.0, 10.9) |
| Female Never Smoker, Male Ever Smoker | 46/77 | 1.3% | 2213 (1962, 2464) | 10.3 (9.6, 11.0) | 12.8 (11.2, 14.6) | 10.2 (8.9, 11.8) |
| Female Ever Smoker, Male Never Smoker | 33/63 | 4.8% | 1939 (1646, 2232) | 9.9 (9.1, 10.7) | 11.3 (9.7, 13.3) | 9.6 (8.1, 11.3) |
| Female and Male Ever Smokers | 25/42 | 11.9% | 2056 (1700, 2412) | 11.0 (10.0, 12.0) | 11.7 (9.7, 14.3) | 9.8 (8.0, 12.1) |

^a^ Data is presented as marginal mean from generalized linear mixed models with random effects, using normal distribution and identity link function. Estimates were calculated adjusting for female age (34.8 years) and BMI (23.8 kg/m^2^), protocol (luteal agonist), educational status (more than college), pack-years history (0.77 packs-years for women; 1.90 packs-years for men) and partner’s smoking status (never smoker).

^b^ Data is presented as marginal mean counts from generalized linear mixed models with random effects, using Poisson distribution and log link function. Estimates were calculated adjusting for female age (34.8 years) and BMI (23.8 kg/m^2^), protocol (luteal agonist), educational status (more than college), pack-years history (0.77 packs-years for women; 1.90 packs-years for men) and partner’s smoking status (never smoker).
