# Supplementary material for: Discrete survival model analysis of a couple’s smoking pattern and outcomes of assisted reproduction
Source: Fertil Res Pract. 2017 Feb 20;3:5. doi: 10.1186/s40738-017-0032-2 (PMC5416813; doi:10.1186/s40738-017-0032-2)
Supplement: Supplementary file 2 — Adjusted estimated probability of cycles ending in live birth by smoking patterns (EARTH Study, N = 225 couples, 354 ART cycles). (DOCX 17 kb) [file 40738_2017_32_MOESM2_ESM.docx]

**Supplemental Table 2.** Adjusted estimated probability of cycles ending in live birth by smoking patterns (EARTH Study, N=225 couples, 354 ART cycles).

|  | **Women/Cycles** | **% of cycles failing prior to embryo transfer** | **Adjusted percent of cycles ending in live birth (95% CI) ^a^** | |
| --- | --- | --- | --- | --- |
| *Female Smoking History* |  |  | **Per embryo transfer** | **Per initiated cycle** |
| Never Smoker | 167/249 | 6.8% | 51.9 (42.2, 61.5) | 47.4 (38.2, 56.7) |
| Ever Smoker | 58/105 | 11.4% | 45.6 (31.3, 60.7) | 39.5 (26.5, 54.2) |
| *Female Smoking History* |  |  |  |  |
| Never Smoker | 167/249 | 6.8% | 51.9 (42.2, 61.5) | 47.2 (38.0, 56.6) |
| Former Smoker | 51/93 | 9.7% | 46.0 (31.3, 61.4) | 40.3 (26.9, 55.4) |
| Current Smoker | 7/12 | 25.0% | 42.5 (13.0, 78.5) | 31.7 (9.5, 67.3) |
| *Couples Ever Smoking Status* |  |  |  |  |
| Female and Male Never Smokers | 121/172 | 7.6% | 52.0 (41.3, 62.4) | 47.5 (37.5, 57.7) |
| Female Never Smoker, Male Ever Smoker | 46/77 | 5.2% | 49.8 (35.0, 64.6) | 45.6 (31.7, 60.2) |
| Female Ever Smoker, Male Never Smoker | 33/63 | 9.5% | 40.9 (25.3, 58.6) | 36.2 (22.0, 53.4) |
| Female and Male Ever Smokers | 25/42 | 14.3% | 52.7 (32.2, 72.2) | 44.3 (26.3, 63.9) |

^a^ Data is presented as marginal mean proportions from generalized linear mixed models with random effects, using binary distribution and logit link function. Estimates were calculated adjusting for female age (34.8 years) and BMI (23.8 kg/m^2^), protocol (luteal agonist), educational status (more than college), pack-years history (0.77 packs-years for women; 1.90 packs-years for men) and partner’s smoking status (never smoker).
